# Personality traits and dimensions of mental health

**DOI:** 10.1038/s41598-023-33996-1

**Published:** 2023-05-01

**Authors:** Weixi Kang, Francois Steffens, Sònia Pineda, Kaya Widuch, Antonio Malvaso

**Affiliations:** 1 Computational, Cognitive and Clinical Neuroimaging Laboratory, Division of Brain Sciences, Department of Medicine, Hammersmith Hospital, Imperial College London, 3rd floor Burlington Danes Building, Du Cane Road, London, W12 0NN UK; 2 University of Pretoria, Pretoria, South Africa; 3 TecnoCampus, Pompeu Fabra University, Barcelona, Spain; 4 University College London, London, UK; 5 Department of Brain and Behavioral Sciences, National Neurological Institute, IRCCS “C. Mondino” Foundation, Pavia, Italy; 6 University of Pavia, Pavia, Italy

**Keywords:** Psychology, Human behaviour

## Abstract

Individuals are different in a relatively constant pattern of thoughts, feeling, and behaviors, which are called personality traits. Mental health is a condition of well-being in which people may reach their full potential and deal effectively with stress, work efficiently, and contribute to their communities. Indeed, the link between personality and mental health as indicated by the 12-item version of the general health questionnaires (GHQ-12) has been well-established according to evidence found by decades of research. However, the GHQ-12 comprises many questions asking about different dimensions of mental health. It is unclear how personality traits relate to these dimensions of mental health. In this paper, we try to address this question. We analyzed data from 12,007 participants from the British Household Panel Study (BHPS) using a confirmatory factor analysis (CFA) and generalized linear models. We replicated the factor structure of GHQ-12 labeled as GHQ-12A (social dysfunction & anhedonia; 6 items), GHQ-12B (depression & anxiety; 4 items), and GHQ-12C (loss of confidence; 2 items). Moreover, Neuroticism was positively related to all dimensions of mental health issues, Extraversion was negatively related to GHQ-12A (social dysfunction & anhedonia) and GHQ-12B (depression & anxiety), Agreeableness and Conscientiousness were negatively related to GHQ-12A (social dysfunction & anhedonia) and GHQ-12C (loss of confidence), and Openness was negatively related to GHQ-12B (depression & anxiety). These results contribute to theories including the predisposition/vulnerability model, complication/scar model, pathoplasty/exacerbation model, and the spectrum model, which propose that personality traits are linked to mental health and explained possible reasons. Psychologists may use results from this study to identify individuals who may be at high risk of developing various non-psychiatric mental health issues and intervene to avoid negative outcomes.

## Introduction

Individuals distinct in a relatively constant pattern of thoughts, feelings, and behaviors, and these differences can be captured by personality traits. Personality traits have been categorized as "essential psychological constructs" are because they have a significant impact on important life aspects of health-related behaviors e.g.,^1,2^, and the likelihood of psychopathology e.g.,^3,4^, crime e.g.,^5^, work experiences e.g.,^6,7^, academic achievement e.g.,^8^, romantic relationships e.g.,^9,10^ and parent–child interaction^11^. Nevertheless, it is unusual for social scientists to find a single domain of interests in which no evidence supporting the importance of personality traits has been presented. Personality psychologists generally believe that there are five major dimensions that can be used to categorize a wide range of possible personality traits. These dimensions are referred to as the Big Five, and they include Extraversion, Neuroticism, Agreeableness, Conscientiousness, and Openness to experience^12^. Extraversion refers to differences among individuals in terms of their friendliness, sociability, level of activity, and experience of positive emotions. Agreeableness refers to differences among individuals in terms of their altruistic behavior, trust, warmth, and friendliness. Conscientiousness refers to differences among individuals in terms of their ability to control their impulses, focus on tasks, and follow rules. Neuroticism refers to differences among individuals in terms of their susceptibility to negative emotions such as anxiety, anger, and sadness. Finally, Openness to experience refers to differences among individuals in terms of their creativity, innovation, and willingness to accept new ideas^13^. The widespread acceptance of the Big Five framework provides a systematic way to define personality differences at the most fundamental levels. This has helped researchers accumulate knowledge about how personality traits are related to various life outcomes.

Psychological health is a significant aspect in total happiness. Mental health, according to the World Health Organization^14^, is "a condition of well-being in which each person fulfills his or her own potential, can cope with typical stressors of life, can work successfully and fruitfully, and can contribute to her or his community". Traditionally, healthcare providers have been able to accurately assess an individual's well-being by looking at their substance misuse, anxiety, distress, and depression^15^. As a result, mental health is described as a state of complete physical, mental, and social well-being rather than the absence of psychiatric diseases^14^. The general health questionnaire (GHQ) is a widely used self-reported questionnaire that has been developed by Goldberg^16^. The GHQ is known for being a reliable indicator of mental health^16–20^. It has been used extensively in different settings, including cross-cultural settings^17,18^, primary health care, and outpatient settings to screen for psychological diseases^16,19,20^. Furthermore, the GHQ has been utilized in demographic research and health assessment surveys^21^.

Recently, many studies began to examine the factor structure of the 12-item version of the GHQ (GHQ-12), although the GHQ-12 was originally created as a unidimensional scale with a few studies use the one-factor latent structure^22,23^. Instead of using a single factor model, other models with 2 or 3 factors have been found to be more suitable. Among these, the 3-factor model has received more empirical support based on research studies^24–28^. Specifically, the three components in the model include the GHQ-12A (social dysfunction and anhedonia; 6 items), GHQ-12B (depression and anxiety; 4 items), and GHQ-12C (loss of confidence; 2 items).

Personality traits has been long linked to psychopathology, as shown by several models including the predisposition/vulnerability model, complication/scar model, pathoplasty/exacerbation model, and the spectrum model^29–32^. Other than psychopathology, it has been proposed personality as a strong predictor of general psychological health^33–35^, which comprises positive mental health/wellbeing^36–38^. Healthy personality development contributes to many areas of well-being and there is a necessity to include personality’s contributions to well-being into current treatments to mental health^39–41^. The five-factor model of personality (FFM) suggests that Neuroticism and Extraversion are the personality traits that are most strongly associated with mental health^42–47^. People who score high on the Neuroticism trait tend to experience negative emotions, respond poorly to stress, and may struggle with impulsivity and psychological distress^48–53^. On the other hand, those who score high on Extraversion tend to enjoy social interactions, feel positive emotions more easily, and have better mental health outcomes^54–67^. However, people who score high on Agreeableness may have worse mental health outcomes, while those who score high on Openness and Conscientiousness may have better outcomes^54,68–70^.

Although many studies have investigated how personality could predict mental health, few studies have investigated how they may relate to dimensions of mental health as mental health is never a unitary concept. To understand how personality traits are associated with dimensions of mental health, we first produce three underlying factors of GHQ-12 and investigate how personality traits are related to dimensions of mental health. We hypothesize that Neuroticism and Extraversion have the strongest positive associations with dimensions of mental health issues whereas other associations between personality traits and dimensions of mental health may vary across dimensions of mental health. Specifically, Agreeableness should be negatively associated with mental health whereas Openness and Conscientiousness are expected to be negatively related to various mental health issues.

## Methods

### Data

We used data from the British Household Panel Study (BHPS)^71^, which is an ongoing longitudinal survey of representative samples of individual households in the UK since 1991. Participants were interviewed in person once a year. The data were collected from September, 2005 to May, 2006 with ethical guidelines following ethical approval by the University of Essex Ethics Committee. This particular dataset was used because it is the only wave that contains personality measures. Informed consent has been obtained from all participants.

### Predictors

BHPS respondents completed an abbreviated 15-item version of the Big Five Inventory^13,72–74^ using a 7- point scale ranging from 1 (‘Does not apply to me’) to 7 (‘Applies perfectly to me’). Each dimension of the Big Five consisted of 3 items. Questions that were used to assess the Big Five personality traits can be found in Table1. Questions optrt5a1, optrt5c2, optrt5e3, and optrt5n3 were reverse coded as these questions were asked in the opposite direction of the corresponding trait. Mean scores were used for each personality traits. The internal consistency analyses revealed the following results: Extraversion (alpha = 0.55), Neuroticism (alpha = 0.68), Conscientiousness (alpha = 0.52), Agreeableness (alpha = 0.53), and Openness (alpha = 0.67). Although these results do not indicate high internal consistency across all five scales, this is not an unusual observation for abbreviated inventories e.g.,^75^. Nevertheless, Donnellan & Lucas^72^ confirmed the 3-item shortened scales were strongly correlated with the full versions of the Big Five Inventory and therefore can be considered as an effective replacement.

**Table 1:** The 15-item version of the BFI, including questions regarding agreeableness, conscientiousness, extraversion, neuroticism, and openness. each dimension of personality consists of three questions.

BFI		
Agreeableness	optrt5a1	I see myself as someone who is sometimes rude to others
	optrt5a2	I see myself as someone who has a forgiving nature
	optrt5a3	I see myself as someone who is considerate and kind to almost everyone
Conscientiousness	optrt5c1	I see myself as someone who does a thorough job
	optrt5c2	I see myself as someone who tends to be lazy
	optrt5c3	I see myself as someone who does things efficiently
Extraversion	optrt5e1	I see myself as someone who is talkative
	optrt5e2	I see myself as someone who is outgoing, sociable
	optrt5e3	I see myself as someone who is reserved
Neuroticism	optrt5n1	I see myself as someone who worries a lot
	optrt5n2	I see myself as someone who gets nervously easily
	optrt5n3	I see myself as someone who is relaxed, handles stress well
Openness	optrt5o1	I see myself as someone who is original, comes up with new ideas
	optrt5o2	I see myself as someone who values artistic, aesthetic experiences
	optrt5o3	I see myself as someone who has an active imagination

### Predicted variables

The GHQ-12 is simple to administer and can be completed by a single participant in less than 10 min^22^. The original GHQ consisted of 60 items and has a number of different versions such as the GHQ-12, GHQ-20, GHQ-28 and GHQ-30. Given its ease of use, the GHQ-12 is one of the most commonly used versions among those listed^23,24^. The GHQ-12 is a self-reported 12-item questionnaire with four indexes for each item. The Likert scoring approach (0–1-2–3) and the bi-modal (0–0-1–1) scoring system are two of the most widely used scoring systems^22^. Banks et al.^25^ have shown the effectiveness of utilizing the GHQ-12 to compare degrees of psychiatric impairment within and between groups. Several studies have validated the psychometric features of this questionnaire^26,27–30^. The GHQ-12 has been demonstrated to have strong specificity, reliability, and reasonably high sensitivity^31,32^. Thus, since Goldberg’s development of the GHQ, it has been used in a variety of countries and cultures, and it has been translated into 38 languages^33–37^. BHPS respondents completed questions asking their age, sex, present legal marital status, highest educational qualification, political party supported, employment status, and questions from the 12-item GHQ (Table2), which used a 7- point scale^22^ ranging from 1 (‘Better than usual’) to 7 (‘Much less than usual’). The internal consistency of the GHQ-12 is 0.90 (alpha = 0.90).

**Table 2:** The GHQ-12 consisting of 12 self-reported questions that assess an individual’s general mental health.

GHQ-12		
oghqa	Have you recently	Been able to concentrate on whatever you're doing?
oghqb	Have you recently	Lost much sleep over worry?
oghqc	Have you recently	Felt that you were playing a useful part in things?
oghqd	Have you recently	felt capable of making decisions about things?
oghqe	Have you recently	Felt constantly under strain?
oghqf	Have you recently	Felt you couldn't overcome your difficulties?
oghqg	Have you recently	Been able to enjoy your normal day-to- day activities?
oghqh	Have you recently	Been able to face up to problems?
oghqi	Have you recently	Been feeling unhappy or depressed?
oghqj	Have you recently	Been losing confidence in yourself?
oghqk	Have you recently	Been thinking of yourself as a worthless person?
oghql	Have you recently	Been feeling reasonably happy, all things considered?

### Analysis

There was data from 15, 617 participants from SHPS Wave 15 in total. Participants who had any missing data field and who were older than 99 or younger than 16 were removed from further analysis because of extremely low numbers of participants in these groups. Thus, a total of 12, 007 data points from participants remained.

### Factor model

Answers from GHQ 12 were taken into a confirmatory factor analysis (CFA) with a specified number of factors 3 in MATLAB 2018a. The three-factor scores for each respondent were computed as the mean of the responses to the items provided by the respondent. Specifically, the three factors were labeled as GHQ-12A (social dysfunction & anhedonia; 6 items), GHQ-12B (depression & anxiety; 4 items), and GHQ-12C (loss of confidence; 2 items).

### Linear models

We examined how Big Five personality traits including Neuroticism, Openness, Agreeableness, Conscientiousness, and Extraversion could predict dimensions of mental health by performing three multiple regressions using demographics and Big Five personality traits including Neuroticism, Openness, Agreeableness, Conscientiousness, and Extraversion and demographics as predictors and GHQ-12A (social dysfunction & anhedonia), GHQ-12B (depression & anxiety), and GHQ-12C (loss of confidence) as predicted variables.

## Results

Descriptive statistics can be found in Table3. The CFA yielded three interpretable factors including GHQ-12A (social dysfunction & anhedonia; 6 items), GHQ-12B (depression & anxiety; 4 items), and GHQ-12C (loss of confidence; 2 items). The loadings of these items can be found in Table4. All the items of the GHQ-12 loaded on the factors they were expected to^24–28^.

**Table 3:** Descriptive statistics of variables of interest.

Variable name	Value	Count (n)	Percent (%)
Sex	Male	7120	45.6
	Female	8497	54.4
Present legal marital status	Married	8115	51.96
	Separated	327	2.09
	Divorced	1248	7.99
	Widowed	1192	7.63
	Never married	4,727	30.27
Highest educational qualification Political party supported	Higher degree	425	2.7
	First degree	1,597	10.2
	Teaching QF	339	2.2
	Other higher QF	3432	22.0
	Nursing QF	161	1.0
	GCE A levels	1811	11.6
	GCE O levels or equi	2517	16.1
	Commercial QF, No O	331	2.1
	CSE Grade 2–5, Scot G	421	2.7
	Apprenticeship	257	1.6
	Other QF	102	0.7
	No QF	2787	17.8
	Still at school No Q	138	0.9
	Conservative	2372	15.2
	Labor	3964	25.4
	Lib Dem/LiB/SDP	1606	10.3
	Scot Nat	396	2.5
	Plaid Cymru	206	1.3
	Green Party	179	1.1
	Other party	142	0.9
	Other answer	54	0.3
	None	2,213	14.2
	Cannot vote	287	1.8
	Ulster unionist	480	3.1
	SDLP	457	2.9
	Alliance party	119	0.8
	Democratice unionist	504	3.2
	Sinn fein	233	1.5
	Other party	42	0.3
Employment status	Self employed	1030	6.60
	In paid employ	7318	46.86
	Unemployed	486	3.11
	Retired	3,074	19.68
	Maternity leave	94	0.60
	Family care	953	6.10
	FT studt, school	877	5.62
	LT sick, disabled	656	4.20
	Govt trng scheme	24	0.15
	Something else	106	0.68

**Table 4:** The factor loadings for the three-factor structure of the GHQ-12.

GHQ-12 items	GHQ-12A (social dysfunction & anhedonia; 6 items)	GHQ-12B (depression & anxiety; 4 items)	GHQ-12C (loss of confidence; 2 items)
Concentration	**0.55**	0.25	− 0.11
Loss of sleep	− 0.03	**0.73**	0.00
Playing a useful Role	**0.73**	− 0.21	0.12
Constantly under Strain	**0.83**	− 0.20	0.05
Problem overcoming difficulties	− 0.10	**0.88**	− 0.05
Unhappy or Depressed	0.04	**0.59**	0.18
Losing Confidence	**0.56**	0.33	− 0.16
Believe worthless	**0.69**	0.03	0.01
General Happiness	0.01	**0.63**	0.24
Capable of making decisions	0.02	0.22	**0.68**
Ability to face Problems	0.09	0.03	**0.75**
Enjoy day− to− day activities	**0.50**	0.21	0.06

The heaviest loading value for each question is in bold.

Demographics and personality traits explained 21.3% (adjusted R2 = 0.213) variances of GHQ-12B (depression & anxiety). Specifically, Neuroticism (β = 0.34; t = 52.05, *p* < 0.001; 95% C.I. [0.33, 0.35]), Extraversion (β = 0.03; t = 3.76, *p* < 0.001; 95% C.I. [0.01, 0.04]) and Openness (β =− 0.02; t = 2.23, *p* = 0.03; 95% C.I. [− 0.04, 0.00]) was positively related to GHQ-12B (depression & anxiety) after controlling for demographics (Table5).

**Table 5:** Estimates (β) of demographics and personality predictors for GHQ-12B (depression & anxiety).

Variables	β	SE	tStat	*p* Value	95% C.I
Intercept	− 1.34	0.08	16.60	< 0.001	[− 1.50, –1.18]
Age	0.00	0.00	0.36	0.72	[0.00, 0.00]
Sex	0.05	0.02	2.70	0.01	[0.01, 0.08]
Present legal marital status	− 0.02	0.01	3.24	0.001	[− 0.03, − 0.01]
Highest educational qualification	0.00	0.00	1.26	0.21	[0.00, 0.01]
Political party supported	0.00	0.00	0.10	0.92	[0.00, 0.00]
Annual income (1.9.2004–1.9.2005)	0.00	0.00	3.15	0.002	[0.00, 0.00]
Employment status	0.01	0.00	2.20	0.03	[0.00, 0.02]
Neuroticism	0.34	0.01	52.05	< 0.001	[0.33, 0.35]
Openness	− 0.02	0.01	2.23	0.03	[− 0.04, 0.00]
Agreeableness	0.01	0.01	0.99	0.32	[− 0.01, 0.02]
Conscientiousness	− 0.01	0.01	1.45	0.15	[− 0.03, 0.00]
Extraversion	0.03	0.01	3.76	< 0.001	[0.01, 0.04]

Demographics and personality traits explained 10.7% (adjusted R2 = 0.107) variances of GHQ-12A (social dysfunction & anhedonia). Specifically, Neuroticism (β = 0.14; t = 19.95, *p* < 0.001; 95% C.I. [0.13, 0.15]) was positively related to GHQ-12A (social dysfunction & anhedonia) whereas Extraversion (β =− 0.07; t =− 9.04, *p* < 0.001; 95% C.I. [− 0.09, –0.06]) Conscientiousness (β = − 0.04; t =− 3.98, *p* < 0.001; 95% C.I. [− 0.05, –0.02]) and Agreeableness (β = − 0.02; t = − 2.46, *p* = 0.01; 95% C.I. [− 0.04, 0.00]) were negatively related to GHQ-12A (social dysfunction & anhedonia) after controlling for demographics (Table6).

**Table 6:** Estimates (β) of demographics and personality predictors for GHQ-12A (social dysfunction & anhedonia).

Variables	β	SE	tStat	*p* Value	95% C.I
Intercept	− 0.59	0.09	− 6.86	< 0.001	[− 0.76, –0.42]
Age	0.01	0.00	19.14	< 0.001	[0.01, 0.01]
Sex	− 0.03	0.02	− 1.46	0.14	[− 0.06, 0.01]
Present legal marital status	0.00	0.01	− 0.58	0.56	[− 0.01, 0.01]
Highest educational qualification	0.09	0.00	2.93	0.003	[0.00, 0.01]
Political party supported	0.00	0.00	1.19	0.23	[0.00, 0.01]
Annual income (1.9.2004–1.9.2005)	0.00	0.00	1.65	0.10	[0.00, 0.00]
Employment status	0.03	0.00	6.52	< 0.001	[0.02, 0.04]
Neuroticism	0.14	0.01	19.95	< 0.001	[0.13, 0.15]
Openness	0.01	0.01	0.87	0.38	[− 0.01, 0.03]
Agreeableness	− 0.02	0.01	− 2.46	0.01	[− 0.04, 0.00]
Conscientiousness	− 0.04	0.01	− 3.98	< 0.001	[− 0.05, –0.02]
Extraversion	− 0.07	0.01	− 9.04	< 0.001	[− 0.09, –0.06]

Demographics and personality traits explained 17.9% (adjusted R2 = 0.179) variances of GHQ-12C (loss of confidence). Specifically, Neuroticism (β = 0.26; t = 38.23, *p* < 0.001; 95% C.I. [0.24, 0.27]) was positively related to GHQ-12C (loss of confidence) and Conscientiousness (β = − 0.10; t = 11.73, *p* < 0.001; 95% C.I. [− 0.12, –0.08]), and Agreeableness (β = − 0.06; t = 7.25, *p* < 0.001; 95% C.I. [− 0.07, –0.04]) were negatively related to GHQ-12C (loss of confidence) after controlling for demographics (Table7)

**Table 7:** Estimates (β) of demographics and personality predictors for GHQ-12C (loss of confidence).

Variables	β	SE	tStat	*p* Value	95% C.I
Intercept	− 0.41	0.08	5.00	< 0.001	[− 0.57, –0.25]
Age	0.00	0.00	4.39	< 0.001	[0.00, 0.00]
Sex	0.02	0.02	1.17	0.24	[− 0.01, 0.06]
Present legal marital status	0.01	0.01	2.86	0.004	[0.01, 0.02]
Highest educational qualification	0.01	0.00	3.82	< 0.001	[0.01, 0.02]
Political party supported	0.00	0.00	1.95	0.05	[0.00, 0.01]
Annual income	0.00	0.00	1.83	0.07	[0.00, 0.00]
Employment status	0.03	0.01	6.50	< 0.001	[0.02, 0.04]
Neuroticism	0.26	0.01	38.23	< 0.001	[0.24, 0.27]
Openness	− 0.01	0.01	1.42	0.16	[− 0.03, 0.01]
Agreeableness	− 0.06	0.01	7.25	< 0.001	[− 0.07, –0.04]
Conscientiousness	− 0.10	0.01	11.73	< 0.001	[− 0.12, –0.08]
Extraversion	− 0.01	0.01	0.66	0.51	[− 0.02, 0.01]

## Discussion

Taken together, the aim of the current study was to investigate how Big Five personality traits are associated with dimensions of mental health as measured by GHQ-12. We used a CFA along with three linear models to replicate the findings of previous studies regarding the three factors present within the GHQ-12 questionnaire. These factors include GHQ-12A (social dysfunction & anhedonia), GHQ-12B (depression & anxiety), and GHQ-12C (loss of confidence). The factor loadings in the current study were found to be consistent with those of previous studies^24–28^, with heavy loadings on corresponding items. This study provided novel findings regarding how personality traits may relate to dimensions of mental health as mental health is never a unitary concept.

We also found that Neuroticism is positively related to all components of mental health including GHQ-12A (social dysfunction & anhedonia), GHQ-12B (depression & anxiety), and GHQ-12C (loss of confidence) after controlling for demographics. People who are neurotic^60^ have more negative effects (e.g., GHQ-12A (social dysfunction & anhedonia), GHQ-12B (depression & anxiety), and GHQ-12C (loss of confidence)). These results are harmonious with previous studies that found Neuroticism is related to low subjective well-being^62^, depressive symptoms, anxiety, mood, and substance abuse disorders^23,47–49^. These results may be explained by the possibility that individuals who are through a depressive or anxiety episode may endorse more of these overlapping Neuroticism items during or following the event. Yet, the link between Neuroticism and outcomes related to public health is more than just a result of overlapping criteria. Several longitudinal research have examined the relationship between the notion of Neuroticism and depressive scores while controlling for shared items and contemporaneous depressed moods^76–78^. Additionally, as a summary of previous research indicates, Neuroticism is strongly positively associated with a variety of mental disorders, including schizophrenia, eating disorders, somatoform disorders, and substance use disorders, as well as physical health issues that are not specifically identified by symptoms that coincide with Neuroticism items. Similarly, Openness was also positively related to GHQ-12B (depression & anxiety) according to previous studies. For instance, it has been suggested that depressed participants showed significantly higher Openness scores than participants without depression^79^. However, a longitudinal study found that change in Openness scores did not relate with the occurrence of or the recovery from any depressive or anxiety disorder^80^. The reasons that our results differ can be explained by the fact that we used different instruments for personality and mental health assessments.

The results showed that Agreeableness is a significant positive predictor of two dimensions of mental health including GHQ-12A (social dysfunction & anhedonia) and GHQ-12C (loss of confidence). While there is little evidence to suggest that Agreeableness is related to social dysfunction and anhedonia, Yu et al. (2020) found a positive relationship between Agreeableness and overall social well-being^81^. Another study found a weak but significant relationship between social anxiety and Agreeableness^82^. Our findings seem to be consistent with these previous studies. Additionally, our study found that Agreeableness is positively related to confidence in older adults^83^, which is in line with the notion that Agreeableness and overconfidence are positively associated^84^. Finally, our findings also support previous research suggesting that Agreeableness is unaffected by depression and anxiety^80^.

Moreover, we found that Conscientiousness is predictive of GHQ-12A (social dysfunction & anhedonia) and GHQ-12C (loss of confidence). Previous research studied the relationship between social dysfunction and Conscientiousness^85–88^. Moreover, it was found that increasing the likelihood of a conscientious and socially stable population could have significant health benefits, despite existing evidence indicates that the causal relationships between Conscientiousness and social dysfunction are multifaceted and complex^85,86,88^. The finding that Conscientiousness is negatively associated with loss of confidence seem to be consistent with previous findings^89^.

Consistent with common beliefs e.g.,^90^, Extraversion was related to GHQ-12A (social dysfunction & anhedonia) and GHQ-12B (depression & anxiety). Several studies investigated the relationship between Extraversion and social dysfunction^87,91,92^. According to previous reviews^91,93^, the negative correlations between Extraversion and depression are primarily due to the aspect of Communal Extraversion, the consensual facet of Liveliness, and NEO Positive Emotions. Changes in Extraversion were also linked to changes in depressive disorder and anxiety disorder status^80^.

Some limitations of the current study should be considered in evaluating the findings presented in this study. First, a brief self-report 15-item scale was used to assess personality traits. As reported by different studies^94–96^, the short BFI measure has already been demonstrated to have good psychometric features, and relatively short questionnaires work reasonably well in personality research^97^. Furthermore, studies like this are always hampered by common method variance, with self-report serving as both the predictors and predicted variables. It is frequently desirable to have observer’s and behavioral data to supplement self-report data, and the methodology could account for a portion of the shared variance. Moreover, the relationships between personality traits and dimensions of mental health can be bi-directional as there might be some innate common underlying genetic factors given the survey questions in the Big Five and GHQ-12 may overlap to some extent. Finally, the data was quite old, which may limit its generalizability as society evolves quickly.

In conclusion, we explored the relationship between personality and each dimension of mental health. Our results suggest when looking at the relationship between mental health using personality and the GHQ-12, it is important to consider the finer-grained detail of what the GHQ-12 is asking. Thus, mental health is not a unitary concept but has many dimensionalities, and personality traits are associated with them differently. These results contribute to theories including the predisposition/vulnerability model, complication/scar model, pathoplasty/exacerbation model, and the spectrum model^29–32^, which propose that personality traits are linked to mental health and explained possible reasons. Psychologists may use results from this study to identify individuals who may be at high risk of developing various non-psychiatric mental health issues and intervene to avoid negative outcomes.

## Data Availability

The study materials and data can be accessed at https://www.iser.essex.ac.uk/bhps/documentation/volb/wave15.
